# APOs as promising prognostic biomarkers: correlation with tumor-infiltrating leukocytes in endometrial cancer

**DOI:** 10.3389/fimmu.2026.1646920

**Published:** 2026-02-16

**Authors:** Lina Zhou, Rencheng Wang, Guiqiang Du, Yupeng Wu, Hui Li, Yinyan He, Zhikang Ye, Jiangdong Xiang

**Affiliations:** 1 Department of Obstetrics and Gynecology, Shanghai General Hospital, Shanghai Jiao Tong University School of Medicine, Shanghai, China; 2 Department of Obstetrics and Gynecology, Affiliated Renhe hospital of Shanghai University, Shanghai, China; 3 Shanghai First Maternity and Infant Hospital, Tongji University School of Medicine, Shanghai, China; 4 Department of Obstetrics and Gynecology, Shanghai Tenth People’s Hospital, Tongji University School of Medicine, Shanghai, China

**Keywords:** apolipoproteins, endometrial cancer, prognostic, biomarkers, tumor-infiltrating leukocytes

## Abstract

**Background:**

Apolipoproteins (APOs) are essentially structural and functional components of lipoproteins, which are composed of 22 members and their effects on certain types of cancer have been studied. However, their roles in endometrial cancer (EC), which is one of the most common malignant tumors in gynecology were unclear and rarely investigated.

**Methods:**

We investigated the expression levels of APOs genes in EC. Furthermore, we explored the roles of APOs in prognostic value, and immune infiltrates in EC patients by using different bioinformatics databases. *In-vitro* experiments were also conducted to evaluate the effect of APOs genes expression on migrant abilities of EC cells.

**Results:**

Nine APO genes (APOC1, APOC2, APOC4, APOD, APOE, APOL3, APOL4, APOLD1, and APOO) were found differently expressed between EC and control tissues by the GEPIA2. However, APOC4 was not included in the subsequent analysis due to its low expression in EC tissues. Moreover, mRNA expression levels of APOs were found correlated with the clinicopathological characteristics of EC, including stage, grade, molecular subgroups, p53 mutant conditions, PTEN mutant conditions, and expression levels of ESR1 and ESR2. Meanwhile higher expression levels of APOs were significantly correlated with better (APOD, APOL3) or poorer (APOC1, APOE, APOLD1) OS. ssGSEA showed 7 TILs in EC which differed significantly from those in adjacent noncancerous tissues were correlated with prognosis of EC patients. The expression levels of both APOD and APOE were positively correlated with all 7 TILs. Finally, western blotting showed that 17β-estradiol (E2) increased APOE protein expression level and reduced APOD protein expression level. Furthermore, APOE was identified to promote the cell migration by scratch assay.

**Conclusion:**

The expression of APOs may be a promising prognostic biomarker and is associated with immune invasion as a potential target for endometrial cancer.

## Highlights

- Endometrial cancer (EC) is a major gynecological malignancy. In 2019, significant numbers of new cases emerged: 63,230 in the US and 84,520 in China, with EC being most common in Shanghai and Beijing. According to the GLOBOCAN 2022 estimates covering 185 countries worldwide, there were 420,242 new cases of EC in 2022, accounting for 2.1% of all cancer cases, with 97,704 associated deaths (1% of all cancer deaths) globally. EC ranks as the 15th most common cancer by incidence and the 19th leading cause of cancer death worldwide.
- Apolipoproteins (APOs) are crucial for lipids transport, and there’s a strong connection between obesity and APOs. Epidemiological investigations have reported potential associations between APOs and multiple cancer types.
- APOs correlated with the clinicopathological characteristics of EC, higher expression levels of APOs were significantly correlated with better (APOD, APOL3) or poorer (APOC1, APOE, APOLD1) OS, and APOE was identified to promote the cell migration.
- The expression levels of APOD or APOE were positively correlated with tumor-infiltrating leukocytes, including central memory T cells, MAIT cells, and Th1 cells, which were related to prognosis.
- The expression of APOs may be a promising prognostic biomarker and is associated with immune invasion as a potential target for endometrial cancer.

## Introduction

Endometrial cancer (EC) is one of the most common malignant tumors in gynecology. According to the GLOBOCAN 2022 estimates covering 185 countries worldwide, there were 420,242 new cases of EC in 2022, accounting for 2.1% of all cancer cases, with 97,704 associated deaths (1% of all cancer deaths) globally. EC ranks as the 15th most common cancer by incidence and the 19th leading cause of cancer death worldwide (1). Especially, in Shanghai and Beijing, EC has the highest incidence of gynecological tumors (2). It is widely accepted that obesity, a common metabolic disorder, has been one of the risk factors for EC. Obesity-related alterations in lipid transport precede and may be related to circulating lipid levels and tissue lipid metabolism (3). Apolipoproteins (APOs) serve as critical mediators responsible for binding and transporting lipids to target tissues throughout the body (3, 4). Accordingly, obesity shares a strong association with APOs.

APOs are inherent structural and functional components of lipoproteins, which bind lipids to form lipoprotein complexes (5, 6). As lipid carriers, APOs are ligands for cofactors of enzymes, cell membrane receptors, and structural components of lipoproteins. Epidemiological investigations have reported potential associations between APOs and multiple cancer types (7). Notably, APOs have been shown to restore antitumor immunity and induce long-term tumor-specific immune surveillance (8). Furthermore, researchers have demonstrated that APOs contribute to enhancing the efficacy of immunotherapy in colorectal cancer (9), while a separate study has shown that APOC1 inhibition remodels the hepatocellular carcinoma tumor immune microenvironment, offering a novel strategy to improve patient outcomes (10).

The human APO gene family is composed of 22 members (4): APOA1, APOA2, APOA4, APOA5, APOB-48, APOB-100, APOC1, APOC2, APOC3, APOC4, APOD, APOE, APOH, APOL1, APOL2, APOL3, APOL4, APOL5, APOL6, APOM, APOO, and APOJ, which are divided into 10 subfamilies (APOA-APOJ). The effects of APOs on cardiovascular disease and even on Alzheimer’s disease have been well studied (6, 11, 12), while their role in cancer has been rarely investigated. Some APOs, such as APOD, APOE and AOPA1, have been shown to inhibit tumor growth in mammary tissues (13), while elevated APOE expression in prostate cancer is associated with poor patient prognosis (14). One study reported the expression of some APOs in EC cells and tissues (15), and another showed that in EC, serum APOA1 is significantly decreased yet enhances CD8+ T-cell antitumor activity via HIF-1α-mediated glycolysis (16). However, the real relationship between APOs and EC needs to be further elucidated.

In our previous study, it was found that artesunate inhibited the proliferation of EC cells by enhancing the cytotoxicity of NK92 cells on EC cells via interactions between CD155 and CD226/TIGIT (17). We further investigated the mechanism of artesunate after treating EC cells with it, and we identified changes in the expression levels of APOs, APOM and APOD by next-generation sequencing. These data suggested that APOs may play a role in EC.

Therefore, the aim of this study was to evaluate the roles of APOs family members in EC via bioinformatics analyses. We prioritized assessing the potential of APOs as prognostic biomarkers or therapeutic targets using multiple databases. Furthermore, we sought to explore one of the underlying molecular mechanisms involving APOs, which may provide evidence for clinicians and a novel basis for predicting the survival of EC patients.

## Materials and methods

### Gene expression profiling interactive analysis 2

The GEPIA web server is a vital and highly quoted resource for analyzing gene expression on the basis of tumor and normal samples in The Cancer Genome Atlas (TCGA, https://portal.gdc.cancer.gov) and Genotype-Tissue Expression (GTEx, https://www.gtexportal.org/home/datasets) databases (18). The GEPIA2 (http://gepia.cancer-pku.cn) was herein used to compare the differential expression levels of APO genes and the survival prediction of immune signatures in EC.

### TNMPlot

TNMplot (19) is an open-source online network tool for comparing gene expressions among normal tissues, tumor tissues, and metastatic tissues. Gene arrays from the Gene Expression Omnibus of the National Center for Biotechnology Information (NCBI-GEO) or RNA-seq data from TCGA, Therapeutically Applicable Research to Generate Effective Treatments (TARGET), and GTEx databases were analyzed. In the present research, TNMplot (https://tnmplot.com) was used to analyze differential gene expression in tumor tissue, normal tissue and metastatic tissue. P < 0.05 was considered statistically significant.

### UALCAN

UALCAN (20, 21) is an integrated interactive network for analysis of OMICS data developed by The University of Alabama at Birmingham Cancer Institute in 2017 and updated in 2022. In UALCAN, researchers can access level 3 RNA-seq data from TCGA. Using about 20,500 protein-coding genes in 33 different tumor types in DRUGBANK, GTEx, and Open Targets, gene expression, gene correlation, survival analysis, promoter methylation and pan-cancer analysis can be performed. In the present study, the clinical data of EC patients, such as disease stage and p53 mutation, were collected from UALCAN (http://ualcan.path.uab.edu/index.html).

### Kaplan-Meier plotter

Kaplan Meier plotter (22) can assess the correlation between the expression and survival data of all genes (mRNAs, miRNAs, proteins) in 21 different tumor types of 30 K+ samples, including gastric cancer, ovarian cancer, lung cancer, and breast cancer. It includes GEO, TCGA, and European Genome-phenome Archive (EGA) databases. In the present study, we analyzed the overall survival/progression-free survival (OS/RFS) of EC patients based on differentially expressed APOs by Kaplan-Meier survival curve (http://kmplot.com/analysis).

### TIMER 2.0

TIMER 2.0 (23) is a web server, providing a more reliable immune infiltration level estimation using cancer genome map (TCGA) or six cutting-edge algorithms. TIMER 2.0 web server facilitates comprehensive analysis and visualization of tumor-infiltrating immune cells. we studied the relationship between tumor-infiltrating immune cells and APOs using TIMER 2.0 web server (http://timer.cistrome.org).

### TISIDB

TISIDB (24) is a portal site that explores the interaction between the tumor and the immune system, and it is based on a variety of databases, such as TCGA, UniProt, GO, DrugBank, etc. TISIDB has completely integrated 4176 records in 2530 copies and reported 988 antitumor immunities. The relationships among the differentially expressed APOs genes, immune signatures (immune types), and clinical characteristics (grade and stage) were developed in TISIDB (http://cis.hku.hk/TISIDB).

### Assessment of the immunological characteristics and prognostic value of the tumor microenvironment

The ESTIMATE algorithm was used to estimate the number of stromal cells and immune cells in malignant tumor tissues, and immune score, stromal score, and estimated score were used to reflect the abundance of immune cells, stromal cell infiltration level, and tumor purity, respectively (25). The infiltration level of 24 immune cells was indicated by enrichment scores based on corresponding markers. Single-sample gene set enrichment analysis (ssGSEA) implemented by “GSVA” R package was employed to calculate enrichment scores (26). The cancer immune cycle is reflection of the anticancer immune response and consists of seven steps. The activity of these steps, determining the fate of tumor cells, was evaluated using ssGSEA (27).

### Flow cytometry analysis of lymphocyte subsets in peripheral blood mononuclear cells

Fresh peripheral whole blood was collected from EC patients and promptly processed within 2 hours. Following centrifugation, the peripheral blood lymphocyte pellet was obtained and resuspended to a final cell concentration of 1×10^6^ cells per 100 μl. The antibodies (**Supplementary Table S1**) were used at a pre-optimized 1:100 (v/v) working concentration (1.0 µl per 100 µl blood sample), and antibody-specific immune cell subset markers are listed in **Supplementary Table S2**. The antibodies and blood sample were incubated in the dark for 15 min with gently stirred, and then, supplemented with 450 µL 1× hemolysins (M-30PCFL; Mindray Inc., New York, NY, USA) and vortexed gently for 5 min of room temperature incubation. Finally, these tubes were placed and tested on a BD FACSCantoTM Flow Cytometer (BD Biosciences, San Jose, CA, USA). The data were analyzed using the FlowJo 10.8.1 software (BD Biosciences).

### Cell culture

Human endometrial Ishikawa cell line (Shanghai Bohu Biological Technology Co., Ltd., China) was cultured in a phenol red-free Dulbecco’s modified Eagle’s medium/F12 medium (Gibco, Waltham, MA, USA) supplemented with 10% carbon-stripped fetal bovine serum (S181-F500; Biowest, Paris, France), 100 µg/ml streptomycin, and 100 units/ml penicillin in the presence of 5% CO_2_, 95% humidity at 37 °C. 17β-estradiol (E2; 10^-8^ M; 50-28-2; Sigma Aldrich, St. Louis, MO, USA) and 0.1% dimethyl sulfoxide (DMSO) (control; Con) for treating Ishikawa cells for 48 h, and the cells of the two group were collected for western blotting.

### Western blotting

Proteins were extracted from treated Ishikawa cells (E2 and Control), which were lysed for 30 min on ice, centrifugation was performed at 10,000 g for 30 min at 4 °C, and the supernatant was removed for analysis. The BCA protein assay kit (Cat. No. 23225; Pierce, Rockford, IL, USA) and the Bradford protein method were used to determine total protein content. Proteins (15 µg) were loaded onto 4% stacked, 10% TrIS-glycine prefab gels, separated by SDS-PAGE, and were then transferred to polyvinylidene difluoride membranes (Bio-Rad Laboratories, Hercules, CA, USA; 100v, 1 h). After washing with TBS, the membranes were sealed and incubated overnight at 4 °C with rabbit anti-human APOE (1:2000, ab271944; Abcam, Cambridge, UK) and rabbit anti-human APOD (1:1000, ab256496; Abcam) antibodies. The membranes were then washed and incubated with peroxidase-labeled goat anti-Rabbit IgG (H+L) antibody (1:25, 074-1506, KPL Co., Ltd., Paris, France). The expression level of glyceraldehyde 3-phosphate dehydrogenase (GAPDH), as a loading control protein, was assessed using horseradish peroxidase (HRP)-labeled murine GAPDH monoclonal antibody (Cat. No. KC-5G4; Aksomics Co., Ltd., Beijing, China). Immunoblots were observed using the enhanced chemiluminescence kit (Cat. No. 34095; Pierce) according to the manufacturer’s instructions. We performed densitometric analysis using ImageJ software (1.53t, NIH, USA).

### Measurement of cell migration by scratch assay

Ishikawa cells (5×10^5^ cells per well) were inoculated into 6-well plates for 12 h. The cells were then incubated overnight. The lentivirus of siRNA targeting APOD (si-APOD; TL314722; OriGene, Rockville, MD, USA) and APOE (si-APOE; TR314721; OriGene), and the corresponding control (negative control; TR30023; OriGene) were transfected with reagents (TF81001; OriGene) for 12 h. The culture was then continued with a fresh medium. After transfection of Ishikawa cells, a stable cell line was established, and quantitative reverse transcription polymerase chain reaction (RT-qPCR) was used to verify its effectiveness. The cell monolayer was scratched using a sterile pipette tip, the floating cells were rinsed off with phosphate-buffered saline (PBS), and the medium was replaced with a fresh medium. Cells were then incubated for 48 h and photographed for the second time. ImagePro PLUS software (Media Cybernetics, New York, NY, USA) was used to analyze and calculate the cell migration distance.

### Statistical analysis

The results of western blotting and flow cytometry were analyzed by SPSS 25.0 software (IBM, Armonk, NY, USA). P < 0.05 was considered statistically significant.

## Results

### Differential expressed APO genes in EC patients

**Figure 1:**
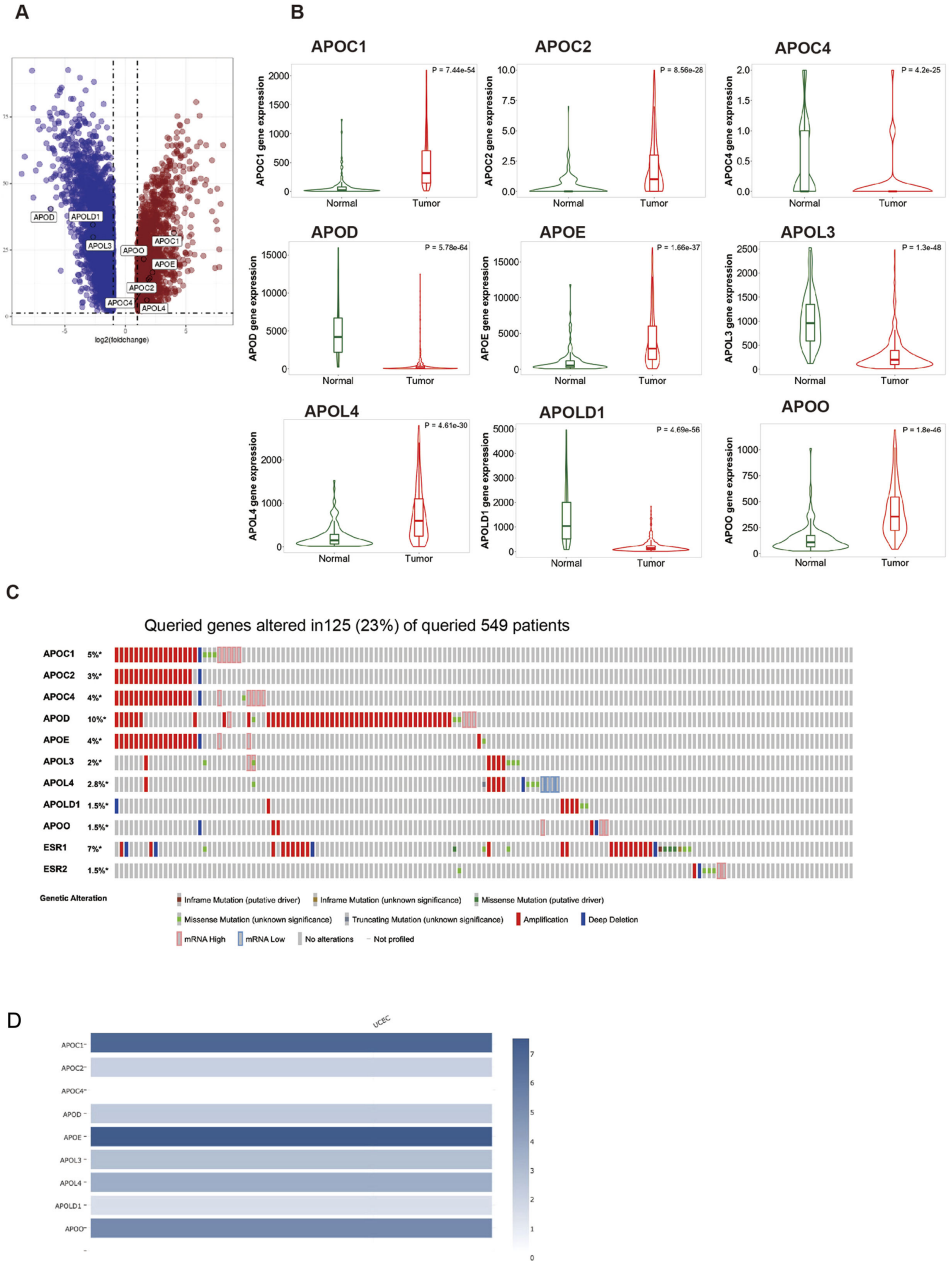
The expression of APOs genes in EC. **(A)** The volcano plots of APOs in EC. Red nodes represent upregulated genes, blue nodes represent downregulated genes, based on the criteria of *p* value < 0.05 and |log2 FC| > 1, respectively. The differential genes were downloaded from GEPIA2, and then the volcano was drawn using Sangerbox tool (http://sangerbox.com/); **(B)** Expression of nine APOs genes in EC and normal tissues in the TNMplot database; **(C)** Genetic alterations of nine APOs genes, ESR1gene and ESR2 gene in EC patients according to cbioportial. **(D)** The relative expression of the APOs in EC from GEPIA2.

First, we analyzed the differential expressed APO genes (APOA-APOJ) in EC and control tissues (TCGA and GTEx databases) by the GEPIA2 (**Figure 1A**). |log2FC|>1 and adjusted P < 0.05 were considered as significant and were selected for subsequent analysis. Totally, 9 differentially expressed APO genes, including APOC1, APOC2, APOC4, APOD, APOE, APOL3, APOL4, APOLD1, and APOO were found. The violin plots were obtained from the TNMPlot database (**Figure 1B**). Then, we queried these differentially expressed APO genes in cBioPortal (https://www.cbioportal.org), and APOD, APOC1, and APOE genes were found in 10%, 5%, and 4% of patients, respectively. At the same time, estrogen receptor 1 (ESR1) gene was identified in only 7% of patients (**Figure 1C**). Although APOC4 had the differential expression between EC and control tissues (**Figure 1B**), and APOC4 was found in 4% of patients (**Figure 1C**), it still very lowly expressed in tissues (**Figure 1D**). Therefore, due to its low expression, APOC4 was not included in the analysis.

### APOs were associated with clinicopathological characteristics of EC

**Figure 2:**
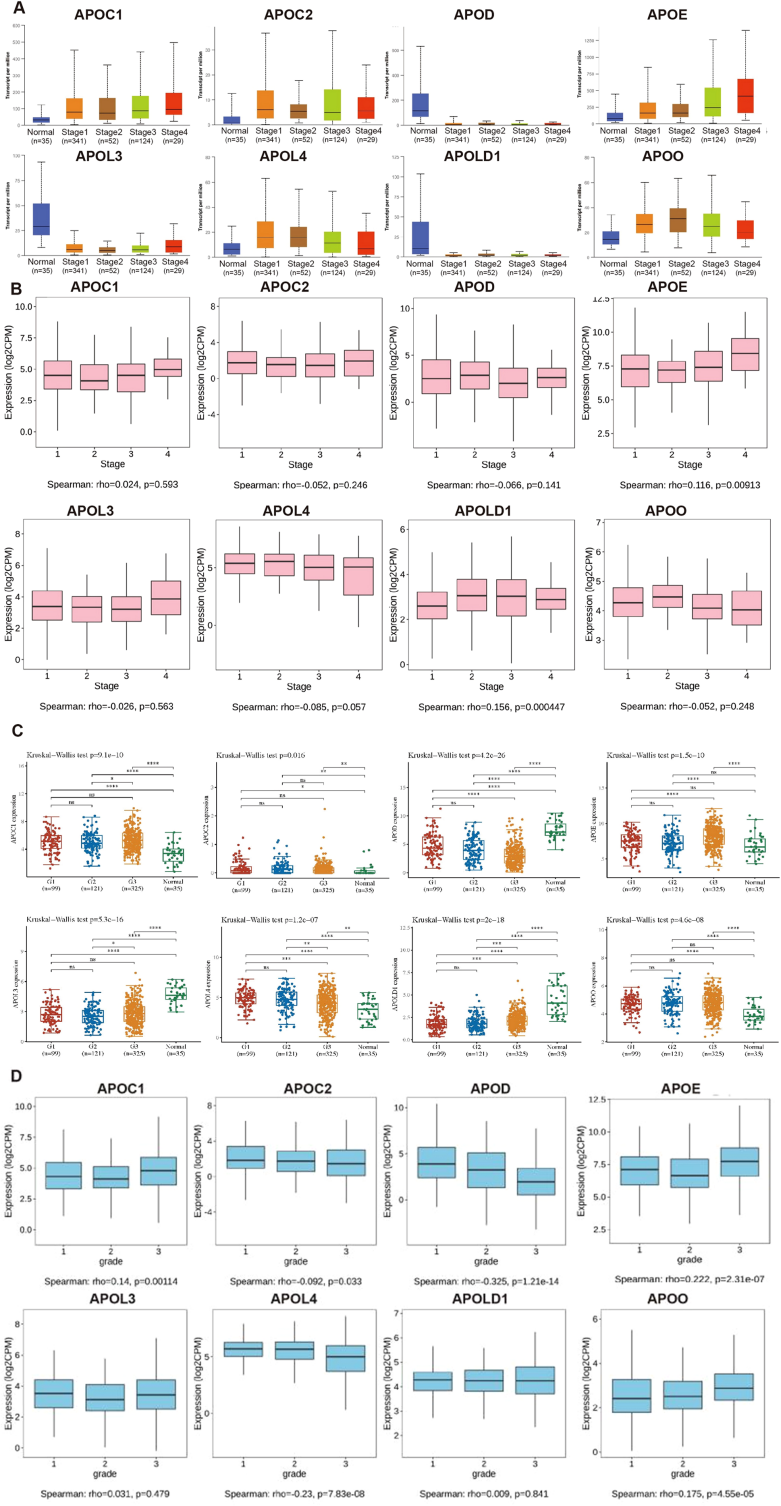
The relationship of the expression level of APOs with clinicopathological conditions in EC. The mRNA expression levels of APOs were analyzed in both normal and different stages **(A)**, different grades **(C)**, the relationships between the APOs and stages **(B)** or grades **(D)** using Spearman correlation in EC. G1: grade 1; G2: grade 2; G3: grade3; Statistical analyses were performed using R software v4.0.3 (R Foundation for Statistical Computing, Vienna, Austria). P < 0.05 was considered statistically significant.

We analyzed the relationships between expression levels of APOs and clinicopathological characteristics (stage and tumor grade) of EC by UALCAN and TISIDB databases (**Figure 2**). APOE expression was significantly higher in tumors than controls across all four stages, with higher mRNA levels positively correlating with advanced stages. Although APOLD1 was lower in EC than controls, it similarly showed a positive correlation with tumor stage. APOD was downregulated in tumors versus controls across all stages but had no significant correlation with stage. Regarding EC grade, APOC1, APOE, and APOO expression positively correlated with grade, while APOC2, APOD, and APOL4 showed a negative correlation.

**Figure 3:**
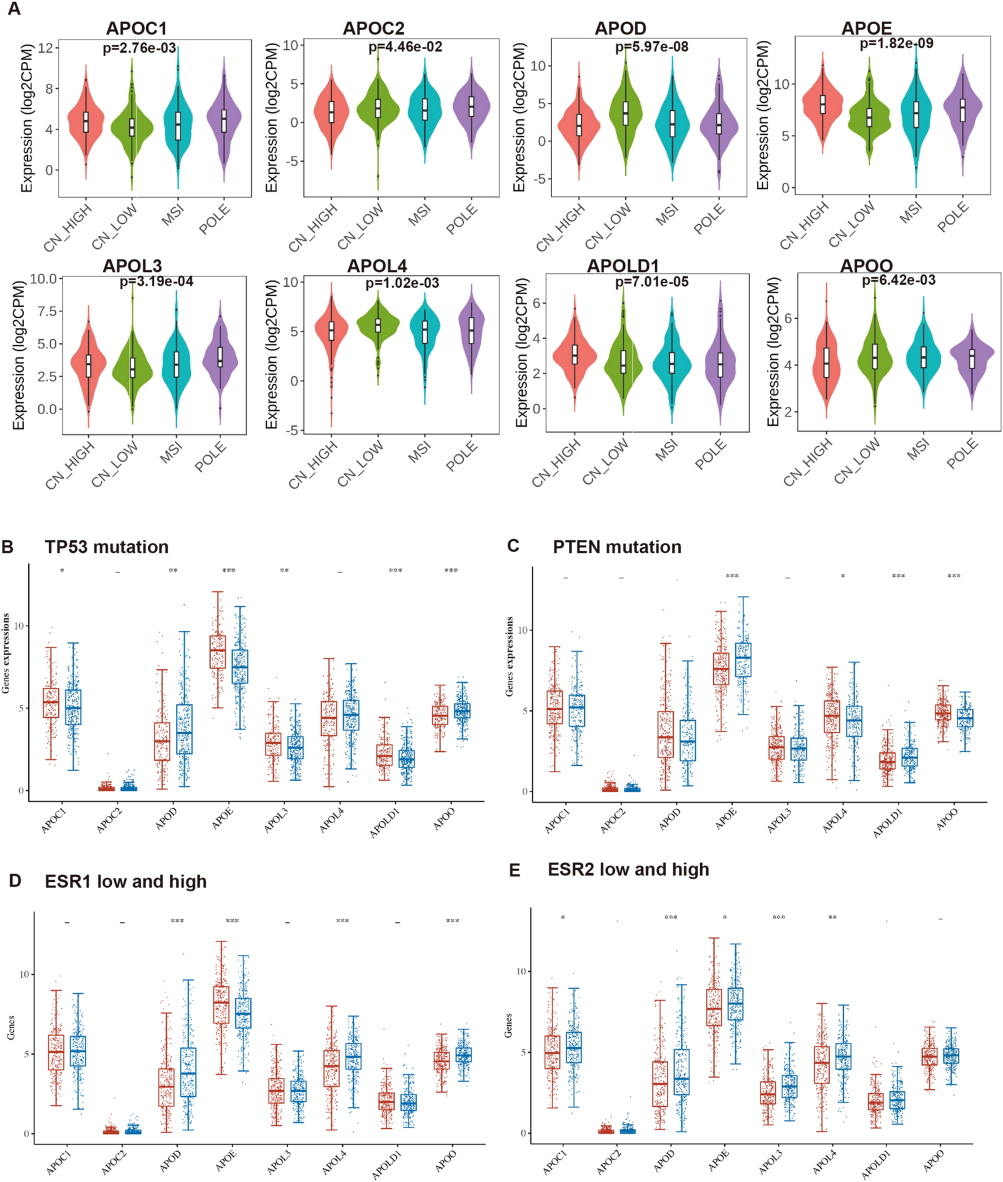
The relationship of the expression level of APOs with molecular characteristics in EC. The mRNA expression levels of APOs were analyzed according to molecular subtypes **(A)**, TP53 mutation **(B)**, PTEN mutation **(C)**, ESR1 expression level **(D)** and ESR2 expression **(E)** level in EC patients. **(A)** from UALCAN database; **(B–E)** from Assistant for Clinical Bionformatics. -: *p* ≥ 0.05, *p < 0.05, **p < 0.01, ***p < 0.001.

In 2013, integrated genomic characterization of EC was established for 4 subgroups: distinct subgroups, POLE (ultra-mutated), microsatellite instability (MSI; hypermutated), low copy number (endometrioid), and high copy number (serous-like) (28). As shown in **Figure 3A**, expression levels of all 8 APOs were significantly differed from different molecular subtypes (high copy number: 160; low copy number: 144; MSI: 124, POLE: 79).

TP53 and PTEN are among the most frequently mutated genes in EC (28, 29), while ESR1 and ESR2 are established EC pathogenic factors. We compared APOs expression between p53-mutant and p53-non-mutant, PTEN-mutant and PTEN-non-mutant, and low and high ESR1/ESR2 expression groups. Results showed APOC1, APOE, APOL3, and APOLD1 were significantly upregulated in p53-mutant EC, whereas APOO and APOD exhibited the opposite pattern (**Figure 3B**). Additionally, PTEN-mutant EC showed higher APOL4/APOO and lower APOE/APOLD1 expression compared with PTEN-non-mutant EC (**Figure 3C**).

Additionally, high expression levels of APOD, APOL4, and APOO were detected in high ESR1 group (**Figure 3D**), and ESR1 expression positively correlated with these three genes (**Supplementary Figure S1A**). In contrast, APOE expression was reduced in the high ESR1 group (**Figure 3D**) and negatively associated with ESR1 (**Supplementary Figure S1A**). Meanwhile, high expression levels of APOC1, APOD, APOE, APOL3, and APOL4 were detected in high ESR2 group (**Figure 3E**). GPR30 (encoded by *GPER1*) is an estrogen transmembrane receptor, and the estrogen/GPR30 signaling pathway in EC has been characterized in our prior work (30, 31). We further evaluated APO expression differences between high and low ESR1 groups, and analyzed their associations with ESR2 and GPER1 (**Supplementary Figures S1**–**S3**).

Therefore, APOs were significantly correlated with clinicopathological characteristics (stage, grade, molecular subgroups, p53/PTEN mutation status, and ESR1/ESR2 expression levels). Of the 8 APOs, only APOE mRNA was significantly associated with all these characteristics.

### Prognostic value of mRNA expression levels of the APO genes in EC patients

**Figure 4:**
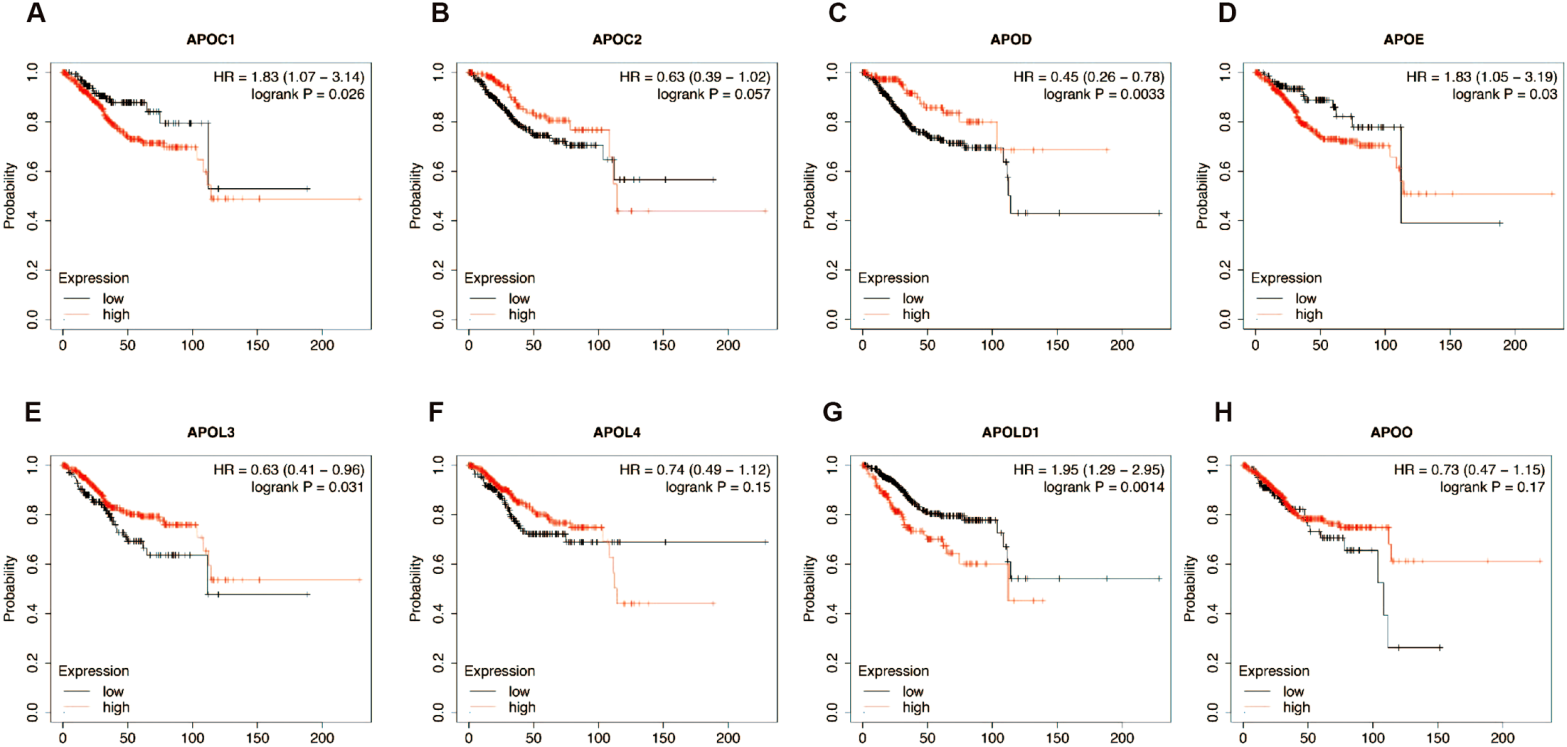
Kaplan–Meier analysis of association between EC prognosis and the expression of APOC1 **(A)**, APOC2 **(B)**, APOD **(C)**, APOE **(D)**, APOL3 **(E)**, APOL4 **(F)**, APOLD1 **(G)** and APOO **(H)**.

The prognostic value of differentially expressed APOs in EC patients was determined by Kaplan-Meier analysis of overall survival (OS) using the Kaplan-Meier plotter (**Figure 4**). According to the results, higher expression levels of APOD and APOL3 were significantly correlated with a longer OS. In contrast, higher expression levels of APOC1, APOE, and APOLD1 were associated with a poor OS. Therefore, APOs expression may considered as predictors for better or poorer OS, indicating that APOs may be promising prognostic biomarkers for EC patients.

APOE showed the strongest correlation with EC risk factors (tumor stage/grade, TP53/PTEN mutation, ESR1/ESR2 expression), followed by APOD (4 factors: tumor grade, TP53 mutation, ESR1/ESR2 expression), APOC1 (3 factors: tumor grade, p53 mutation, ESR1 expression), and APOLD1 (3 factors: tumor stage, p53/PTEN mutation). Thus, among the 8 APOs, APOE, APOD, APOC1, and APOLD1 may be the most clinically relevant in EC.

### Tumor-infiltrating leukocytes from TCGA database analyzed by ssGSEA and partly verified in EC patients

**Figure 5:**
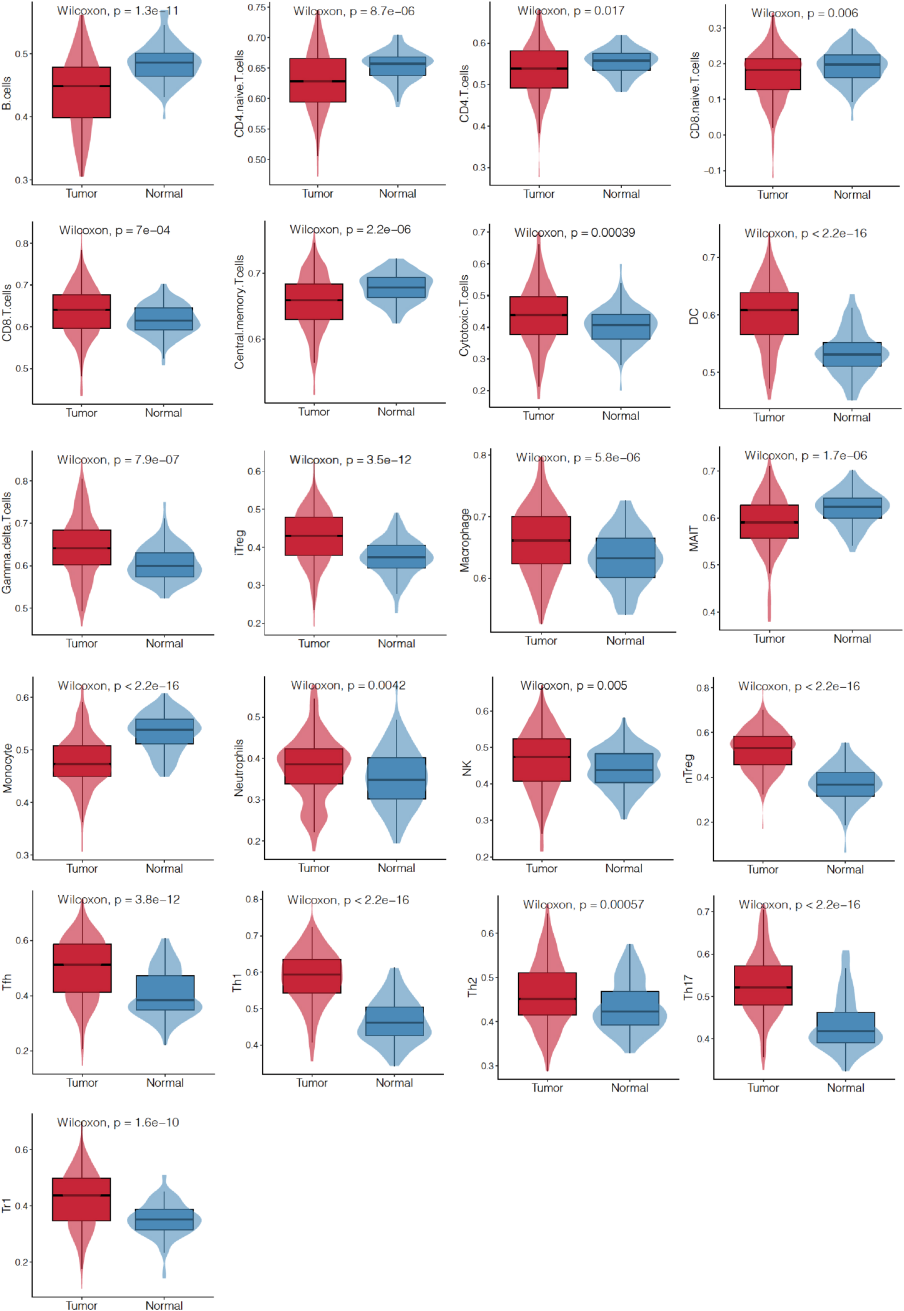
Differences of TILs between EC and those adjacent noncancerous tissues from TCGA database by ssGSEA. Infiltration levels of 24 kinds of TILs were analyzed and 21 of them showed different abundance between the two tissues. TILs, tumor-infiltrating lymphocytes.

TME is closely associated with cancer prognosis and therapeutic effects. TILs were found as integral components of the TME and were correlated with prognosis and therapeutic response. Then, we analyzed the differences of TILs between EC and adjacent noncancerous tissues by ssGSEA. It was found that 21/24 (87.5%) of TILs in EC differed significantly from those in adjacent noncancerous tissues (**Figure 5**).

**Figure 6:**
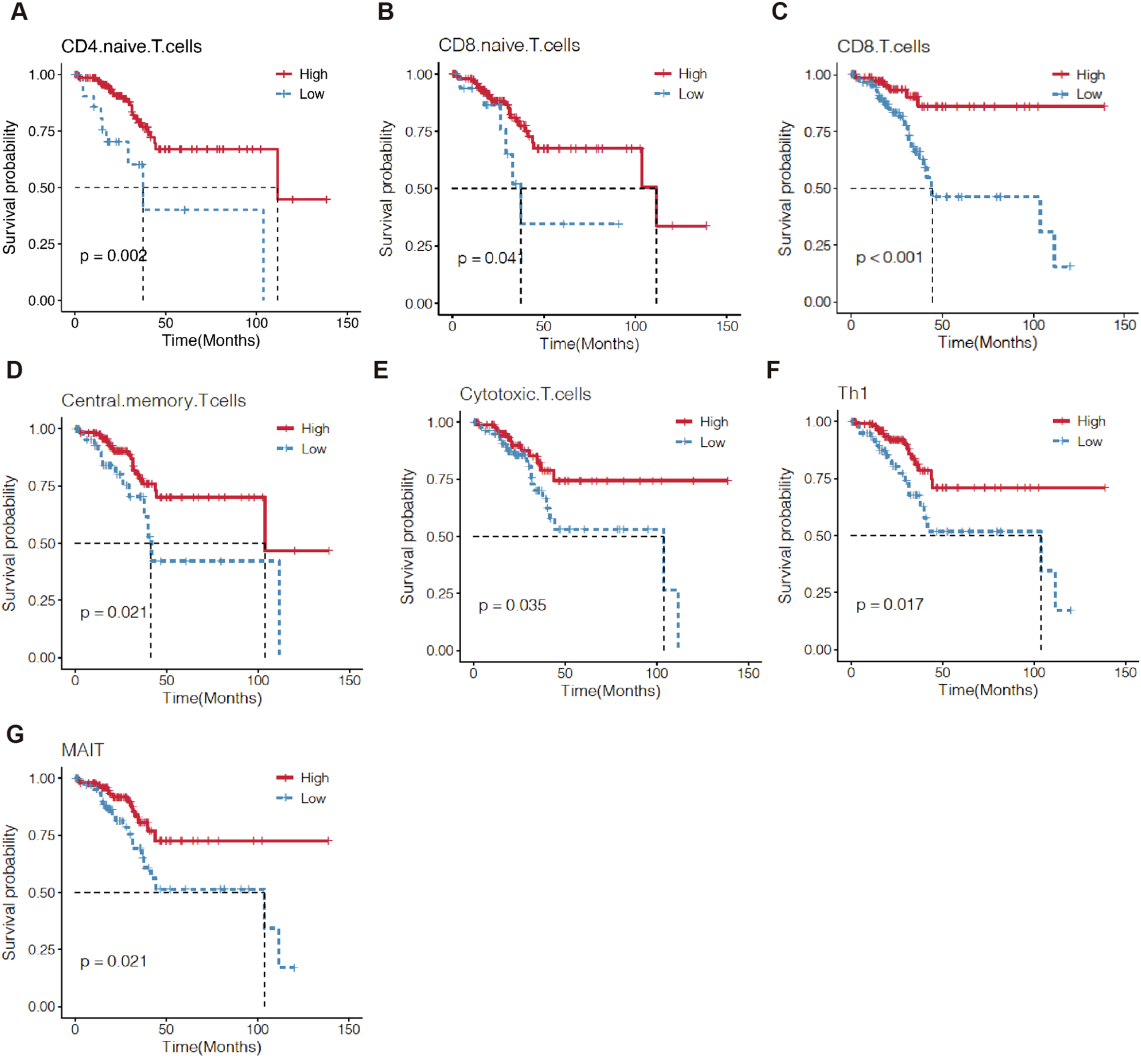
Kaplan–Meier survival curves comparing the high and low level of TILs in EC by ssGSEA. The level of 7 TILs, including CD4 naive T cells **(A)**, CD8 naive T cells **(B)**, CD8+T cells **(C)**, central memory T cells **(D)**, cytotoxic T cell **(E)**, Th1 **(F)** and MAIT **(G)** were correlated with OS of EC patients. TILs, tumor-infiltrating lymphocytes; OS, overall survival.

Next, we investigated the prognostic value of TILs. The Kaplan-Meier analysis revealed that EC patients with high CD4+ naïve T cells, CD8+ naïve T cells, CD8+T cells, central memory T cells, cytotoxic T cells (CD3^+^CD8^+^CD28^+^T cells, Tc), mucosal-associated invariant T (MAIT) cells, and T helper type 1 (Th1) cells were correlated with a longer OS (**Figure 6**).

**Figure 7:**
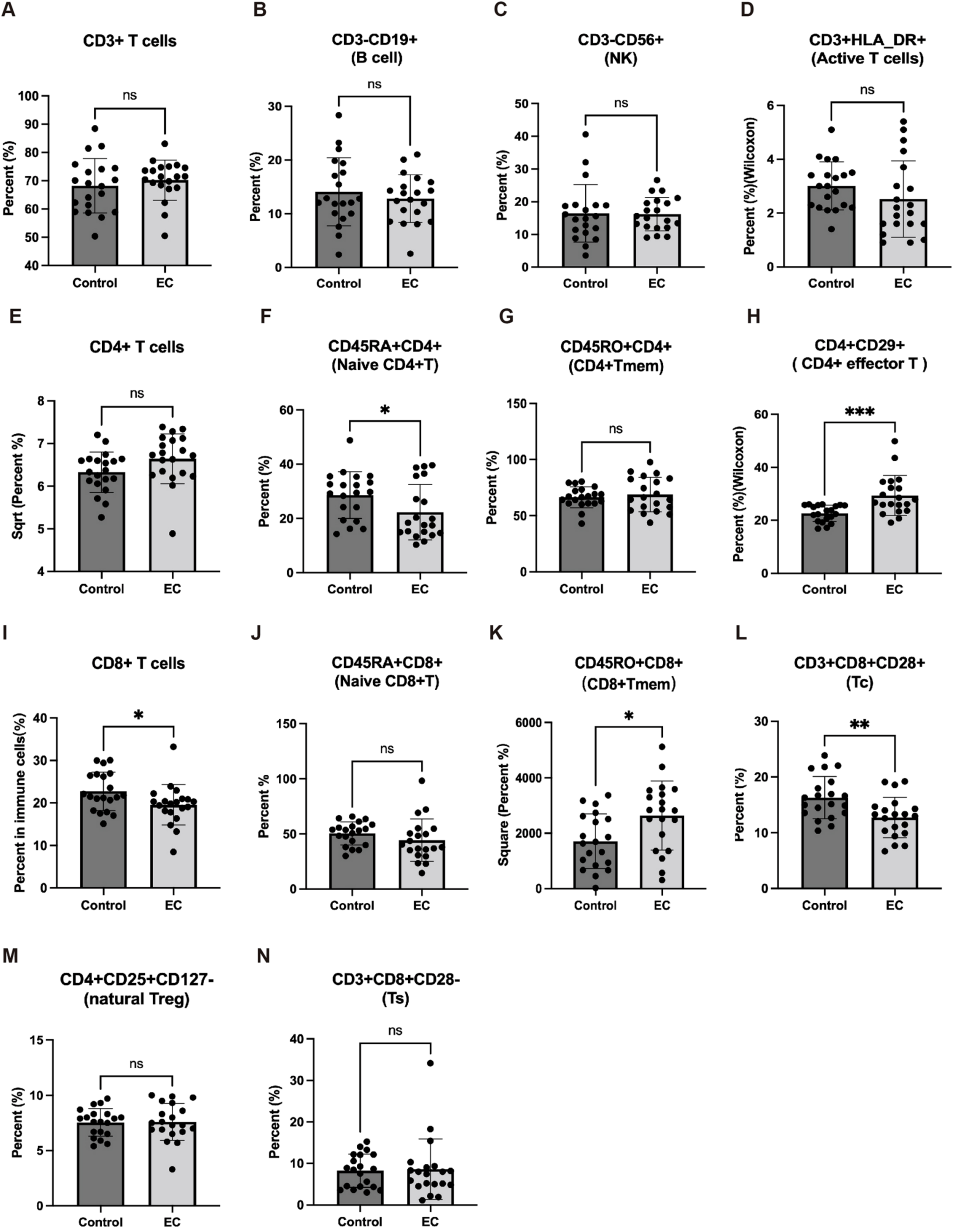
The immune cells in EC patients from peripheral blood mononuclear cell (PBMC). The levels of CD3+ T cells **(A)**, B cell **(B)**, NK cell **(C)**, active T cells **(D)**, CD4+ T cells **(E)**, CD4+ naïve T **(F)**, CD4+ Tmem **(G)**, CD4+ effector T cells **(H)**, CD8+T cells **(I)**, CD8+ naïve T cells **(J)**, CD8+ Tmem **(K)**, cytotoxic T cell (CD3+CD8+CD28+T cell, Tc) **(L)**, nature Treg **(M)**, Ts **(N)** in serum were detected in 20 EC patients and 20 patients with uterus benign myoma. ns*: no significance;* * *p* < 0.05, ** *p* < 0.01.

Finally, we compared the differences of 14 TILs in a detection panel, including CD4+ naïve T cells, CD8+T cells, CD8+ naïve T cells, and Tc in EC patients (20 cases) to control uterine fibroids (controls; 20 cases) (**Figures 7A–N**). We found that CD4+ naïve T, CD8+ T cells, and Tc in EC patients were significantly lower than controls (**Figures 7F, I, L**), however, there was no significant difference in CD8+ naïve T cells between EC and control groups (**Figure 7J**).

### APOD and APOE were correlated with TILs

**Figure 8:**
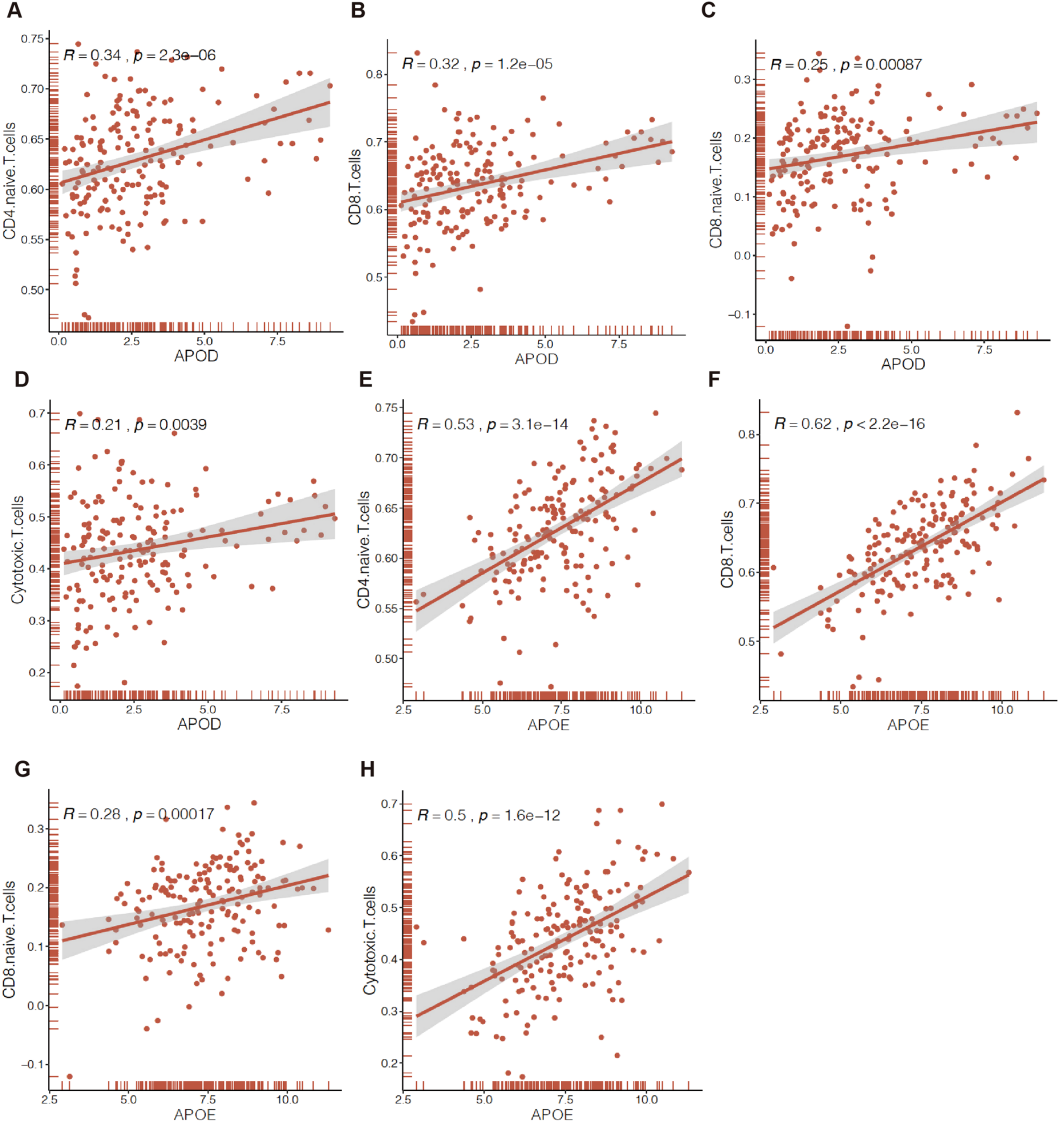
Correlation of APOs expression with immune infiltration in EC by ssGESA. APOD or APOE expression were positively correlated those tumors infiltrating immune cells of CD4+ naïve T cells (CD4 Naïve T) **(A, E)**, CD8+T cells **(B, F)**, CD8+ naïve T cells (CD8 Naïve T) **(C, G)**, cytotoxic T cell (CD3+CD8+CD28+T cell, Tc) **(D, H)**.

We further explored the relationship between the expression levels of APOE or APOD and the level of immune cell infiltration, which would be correlated with prognosis, using Spearman correlation analysis. The results showed that the expression levels of both APOD and APOE were positively correlated with TILs of CD4+ naïve T cells (**Figures 8A, E**), CD8+T cells (**Figures 8B, F**), CD8+ naïve T cells (**Figures 8C, G**), and Tc (**Figures 8D, H**). Other TILs, including central memory T cells, MAIT cells, and Th1 cells, which were related to prognosis, were also positively correlated with the expression levels of APOD or APOE (**Supplementary Figure S4**).

### Estrogen-mediated regulation of APOE and APOD modulated the migratory capacity of endometrial cancer cells

**Figure 9:**
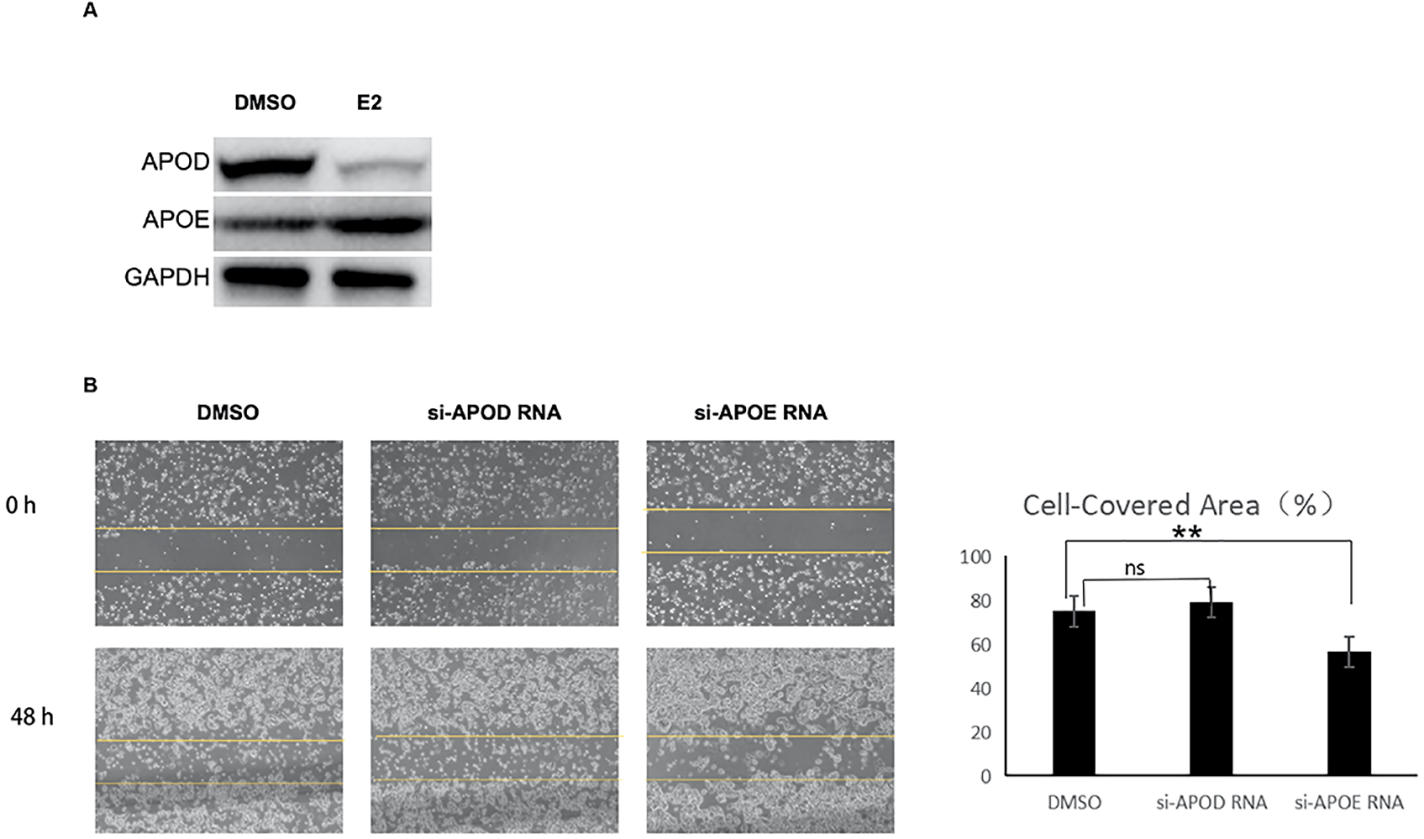
APOE and APOD regulated by estrogen modulated the migration of endometrial cancer cell. **(A)** Western blotting analysis of APOD and APOE in ISHKAWA cells treated with control (DMSO, solvent of E2) or 10^-8^mol/L E2 for 48 hours; **(B)** Wound healing assay was performed to assess cell migration. Cells were transfected with control (DMSO), si-APOD RNA and si-APOE RNA. Migratory cells were observed using an IX711 phase contrast microscope (Olympus Corporation) at 0 and 48 h (magnification, ×200). E2: 17β-estradiol; si-APOD RNA: lentivirus of siRNA targeting APOD; si-APOE RNA: lentivirus of siRNA targeting APOE. ns*: no significance;* **p < 0.05.

To clarify the regulatory effects of E_2_ on APOE and APOD expression, we first detected the protein levels of these two genes in Ishikawa cells treated with or without E_2_. As shown in **Figure 9A** and **Supplementary Figure S5**, E_2_ stimulation significantly upregulated the protein expression of APOE (0.072 ± 0.003 *vs* 0.056 ± 0.009, p=0.0298), whereas it exerted an inhibitory effect on APOD protein expression (0.027 ± 0.007 *vs* 0.061 ± 0.005, p=0.0003). Furthermore, *in vitro* wound healing assay confirmed that APOE promoted the migratory potential of EC cells (**Figure 9B**).

## Discussion

APO family members have been widely recognized as key regulators in the oncogenesis and progression of multiple malignancies. For instance, APOA1 is upregulated in small cell lung cancer, hepatocellular carcinoma, and bladder cancer, and its high expression correlates with tumor recurrence and poor prognosis (4). APOC1 is overexpressed in pancreatic cancer, and the increased serum APOC1 level is associated with a poor prognosis. Knockout of APOC1 suppresses the proliferation of pancreatic cancer cells and promotes cell apoptosis (4, 7). In contrast, APOD mRNA expression is significantly downregulated in both colorectal cancer and hepatocellular carcinoma (HCC) tissues relative to their normal counterparts, and reduced APOD expression is associated with shortened OS (7). APOE is essential for the survival and growth of ovarian cancer cells due to its overexpression (4, 7). These findings collectively demonstrate the multiple functional roles of APOs in cancer, laying the foundation for exploring their correlation in EC.

Despite the well-documented oncologic roles of APO family members in multiple malignancies, their functional relevance in EC remains largely understudied, creating a critical knowledge gap in EC pathogenesis research. The present study fills this gap by systematically analyzing the clinical significance and expression levels of APOs in EC tissues and cells through bioinformatic analysis, and supplemented by partial experimental validation. We totally identified 9 differentially expressed APO genes between EC and normal endometrial tissues. Among these, APOD, APOC1, and APOE genes were found in 10%, 5%, and 4% of patients, respectively, suggesting their potential involvement in EC pathogenesis. Moreover, five APO genes (APOD, APOL3, APOE, APOC1, and APOLD1) were found to be correlated with EC prognosis, with APOE showing the strongest association with EC risk factors (tumor stage, tumor grade, TP53 mutation, PTEN mutation, ESR1/ESR2 expression level), followed by APOD (correlated with 4 factors) and APOC1/APOLD1 (correlated with 3 factors each). These results indicating that APO family members may serve as key modulators of EC progression.

TILs are core components of the TME and play pivotal roles in tumor growth and progression. Previous studies have identified M0 macrophages and CD8+ T cells as prognostic factors for EC (32), and demonstrated that EC are frequently infiltrated by tumor-reactive TILs (33). A high regulatory T cell (Treg) count was associated with poor disease-free survival in EC patients, while stromal CD3+ T cells exhibited prognostic value (34). Consistent with these findings, our ssGSEA analysis revealed significant differences in 7 TIL subsets (CD4+ naïve T cells, CD8+ T cells, CD8+ naïve T cells, Tc cells, central memory T cells, MAIT cells, and Th1 cells) between EC tissues and adjacent noncancerous tissues, with high levels of these TILs correlating with longer OS in EC patients. Validation in peripheral blood mononuclear cells (PBMCs) from 20 EC patients and 20 controls showed that EC patients had significantly lower counts of CD4+ naïve T cells, CD8+ T cells, and Tc compared to controls, although no significant difference was observed in CD8+ naïve T cells. While discrepancies in TIL counts between PBMCs and local tumor tissues may exist due to the tissue-specific nature of TME, our results revealed the important role of the 3 TILs in EC to some extent. It could be speculated that a high level of these TILs in EC patients may reflect better outcomes, while further research is required to confirm this finding.

The crosstalk between APOs and TILs in tumor immunity has been rarely reported, representing a novel research direction. Prior studies have shown that APOE decreases intratumoral CD8+ T cell levels to promote immune suppression in pancreatic ductal adenocarcinoma (35), and APOB downregulation affects immune cell infiltration in cholangiocarcinoma (36). More broadly, APOs are known to influence anti-tumor immunosurveillance (8) and enhance the cytotoxic capacity of CD8+ T cells (9). Building on these findings, our study demonstrated that APOD and APOE (the APO members most strongly associated with EC) exhibited positive correlations with CD4+ naïve T cells, CD8+ T cells, and Tc—TIL subsets closely linked to EC prognosis. These findings indicated that APOs may modulate EC progression by regulating TIL infiltration and function, providing a new mechanistic link between APOs and EC immune regulation that has not been previously reported.

Estrogen signaling pathway activation is a well-recognized key driver of EC occurrence and progression. Our *in vitro* experiments using the Ishikawa cell line showed that E_2_ stimulation significantly upregulated the protein expression of APOE, whereas it suppressed the protein expression of APOD. APOE upregulation promoted cell migration, while APOD downregulation inhibited cell migration. These results not only confirm the functional involvement of APOs in EC progression but also further highlight the functional heterogeneity of APO family members in EC: APOE, which is overexpressed in EC tissues, correlates positively with tumor stage, tumor grade, and p53 mutation, and negatively with OS, suggesting an oncogenic role; in contrast, APOD, which is downregulated in EC tissues, correlates negatively with tumor stage, tumor grade, and p53 mutation, and positively with OS, indicating a tumor-suppressive role. Notably, both APOD and APOE were positively correlated with CD4+ naïve T cells, CD8+ T cells, and Tc (subsets associated with longer OS), which appears contradictory to the oncogenic role of APOE. This inconsistency may reflect the complexity of tumor pathogenesis, in which APOE exerts dual effects in EC by simultaneously promoting tumor malignant phenotypes and regulating antitumor immunity, and the net effect of these dual roles determines clinical outcomes. Such dual roles have been reported for other oncogenes in solid tumors, emphasizing the need for comprehensive mechanistic exploration. Collectively, these results demonstrated that APOs may have a potential value as a prognostic biomarker and therapeutic target for EC.

Despite the novel insights gained, this study has several limitations that need to be acknowledged. First, the study relied heavily on bioinformatic analyses of public databases, with limited experimental validation. In particular, *in vivo* validation is lacking—we only performed *in vitro* experiments using a single EC cell line (Ishikawa), leading to potential single-cell line bias that may limit the generalizability of our findings. Second, mechanistic exploration is limited: while we identified correlations between APOs and TILs/EC risk factors, the underlying molecular mechanisms remain unclear. Third, immune cell analysis was restricted to PBMCs rather than TME-resident cells, which cannot fully reflect the actual state of TIL infiltration in the EC microenvironment, potentially introducing biases in the assessment of APO-TIL crosstalk. Fourth, the retrospective nature of the study, based on public database data, leads to inherent limitations such as unstandardized interventional measures and missing detailed clinical information, which may introduce confounding factors and affect the reliability of prognostic analyses.

To address the above limitations and extend our findings, we propose the following testable future directions: first, establish *in vivo* xenograft to validate the effects of APOE overexpression/APOD downregulation on EC tumor growth and TIL infiltration, complementing *in vivo* evidence; second, perform multi-cell line experiments to eliminate single-cell line bias; third, conduct in-depth mechanistic studies to dissect APO-mediated TIL regulation pathways and their crosstalk with estrogen signaling, fourth, carry out prospective clinical studies with larger EC cohorts to validate APO prognostic value and TIL correlation, incorporating detailed clinical data to reduce confounding biases.

## Conclusion

Our study systematically explores the expression profiles, clinical significance, and immune regulatory roles of APO family members in EC. The findings suggest that APOs (especially APOE and APOD) may serve as potential prognostic biomarkers and therapeutic targets for EC. Subsequent studies addressing the current limitations will further clarify the value of APOs in the diagnosis and treatment of EC.

## Data Availability

The original contributions presented in the study are included in the article/**Supplementary Material**. Further inquiries can be directed to the corresponding authors.

## Supplementary material

Supplementary Figure 1 The relationship between APOs and ESR1 expression. From cbioportal database [cbioportal/Uterus/Endometrial Carcinoma/Uterus Corpus Endometrial Carcinoma (TCGA,Firehose Legacy, 549samples)]

Supplementary Figure 2 The relationship between APOs and ESR2 expression. From cbioportal database [cbioportal/Uterus/Endometrial Carcinoma/Uterus Corpus Endometrial Carcinoma (TCGA,Firehose Legacy, 549samples)]

Supplementary Figure 3 The relationship between APOs and GPER1 expression. From cbioportal database [cbioportal/Uterus/Endometrial Carcinoma/Uterus Corpus Endometrial Carcinoma (TCGA,Firehose Legacy, 549samples)]

Supplementary Figure 4 Some immune infiltration cells correlation with APOD or APOE in EC by ssGESA. APOD or APOE expression were positively correlated the tumor infiltrating immune cells of Central Memory T cells **(A, D)**, MAIT (Mucosal Associated Invariant T, MAIT) **(B, E)** and Th1**(C, F)**.

Supplementary Figure 5 Uncropped original Western blotting images.
