# Supplementary figures and images for: APOs as promising prognostic biomarkers: correlation with tumor-infiltrating leukocytes in endometrial cancer
Source: Front Immunol. 2026 Feb 16;17:1646920. doi: 10.3389/fimmu.2026.1646920 (PMC12950738; doi:10.3389/fimmu.2026.1646920)

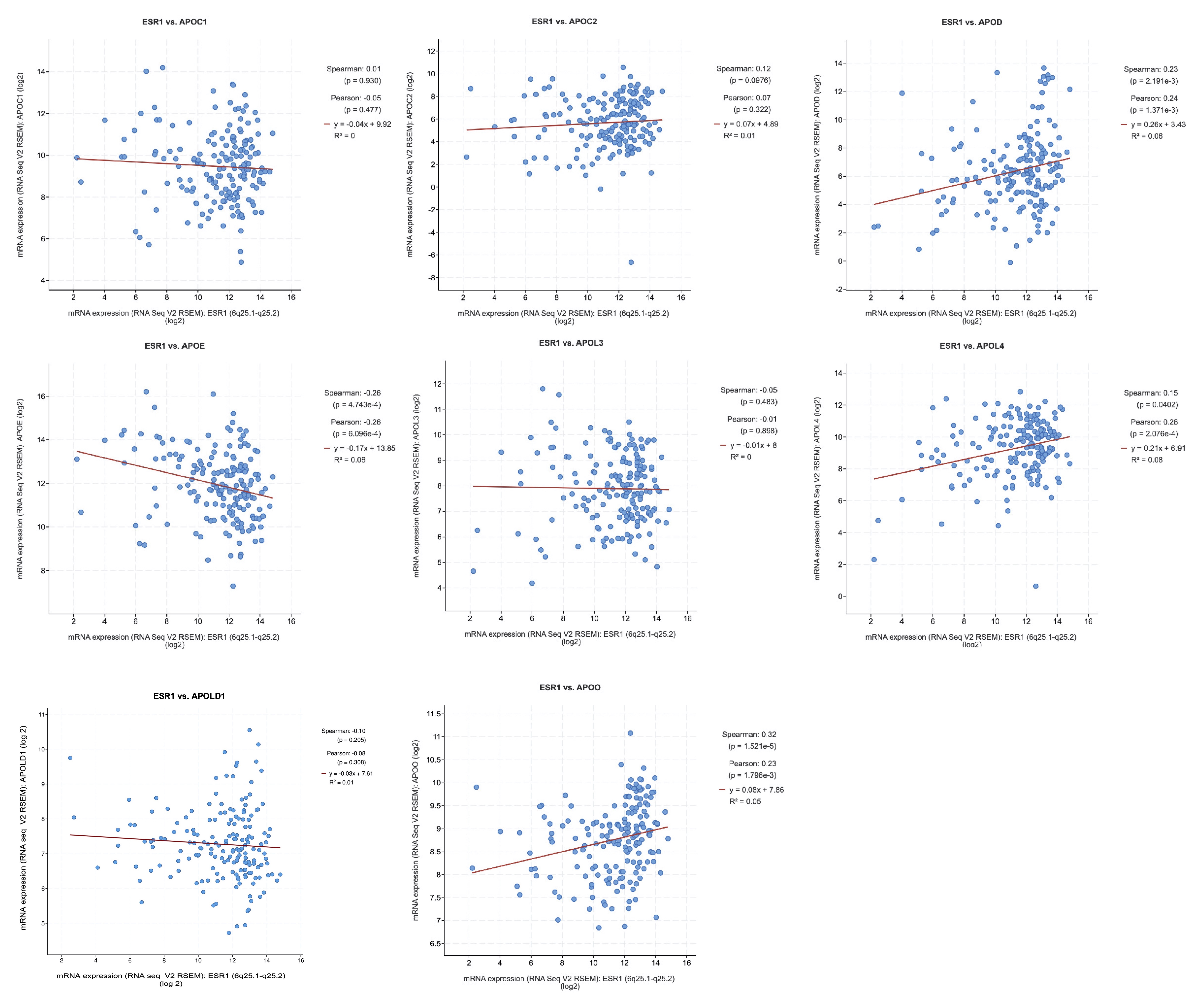

Supplement: Supplementary Figure 1 — The relationship between APOs and ESR1 expression. From cbioportal database [cbioportal/Uterus/Endometrial Carcinoma/Uterus Corpus Endometrial Carcinoma (TCGA,Firehose Legacy, 549samples)] [file Image1.jpeg]

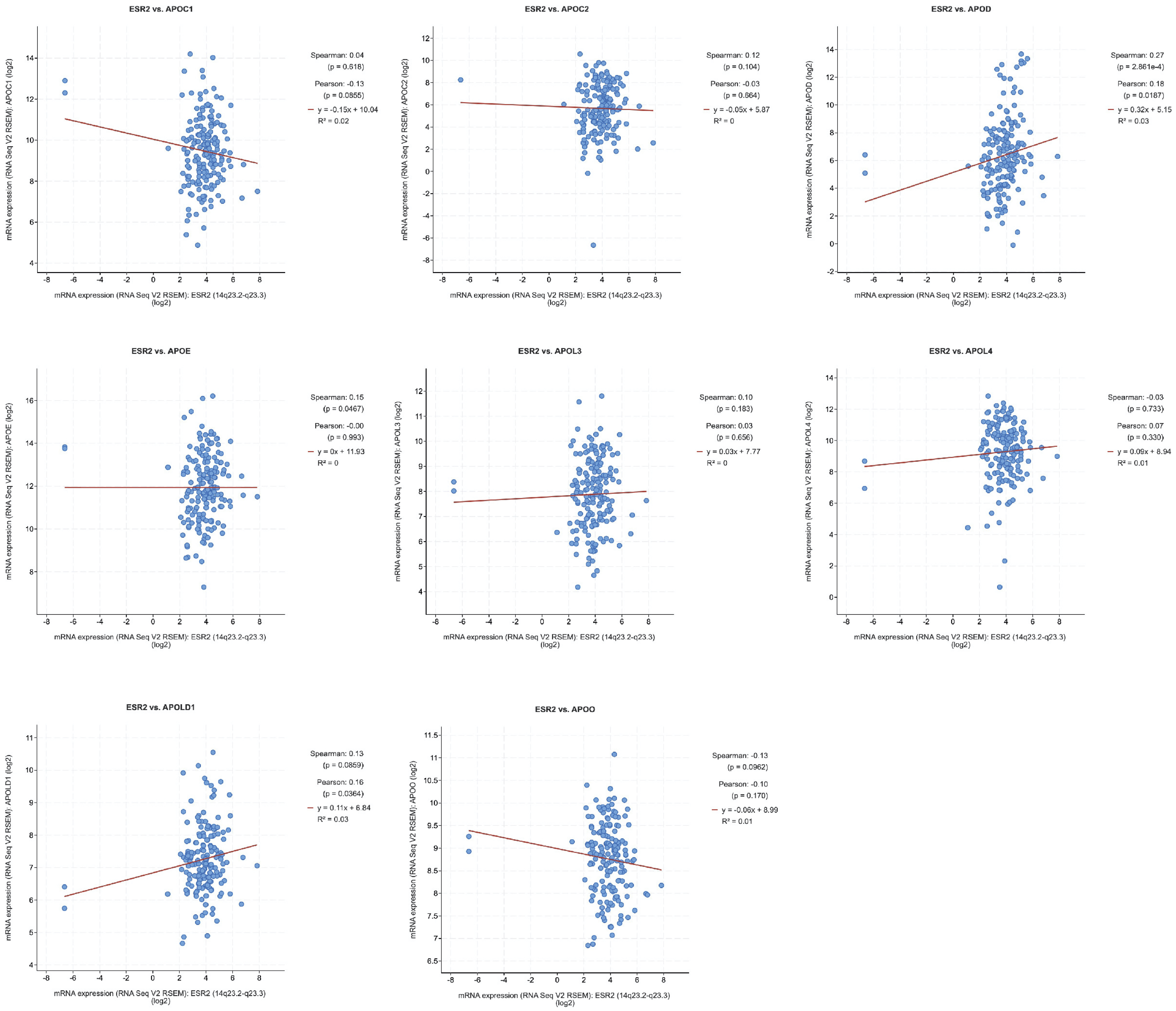

Supplement: Supplementary Figure 2 — The relationship between APOs and ESR2 expression. From cbioportal database [cbioportal/Uterus/Endometrial Carcinoma/Uterus Corpus Endometrial Carcinoma (TCGA,Firehose Legacy, 549samples)] [file Image2.jpeg]

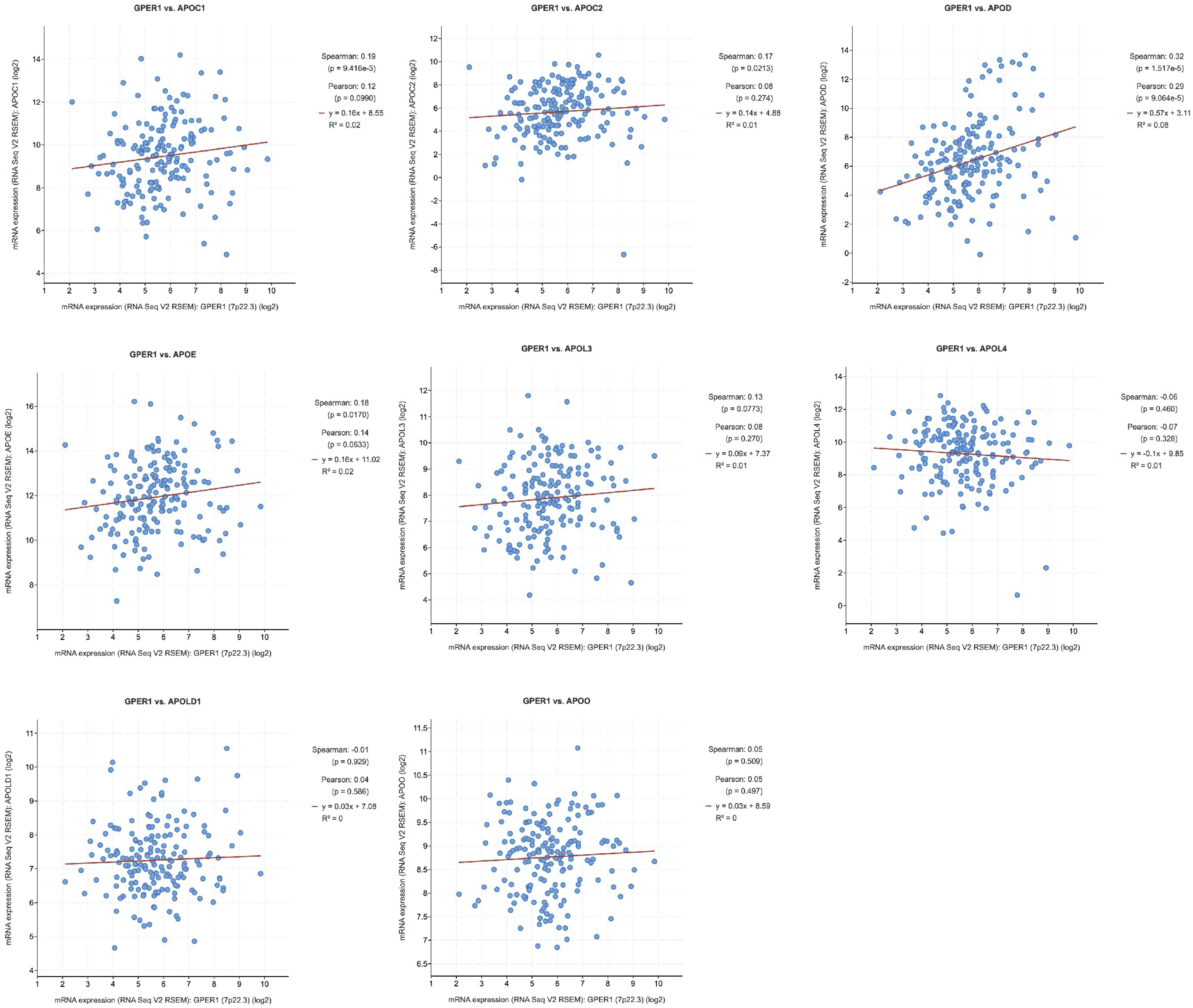

Supplement: Supplementary Figure 3 — The relationship between APOs and GPER1 expression. From cbioportal database [cbioportal/Uterus/Endometrial Carcinoma/Uterus Corpus Endometrial Carcinoma (TCGA,Firehose Legacy, 549samples)] [file Image3.jpeg]

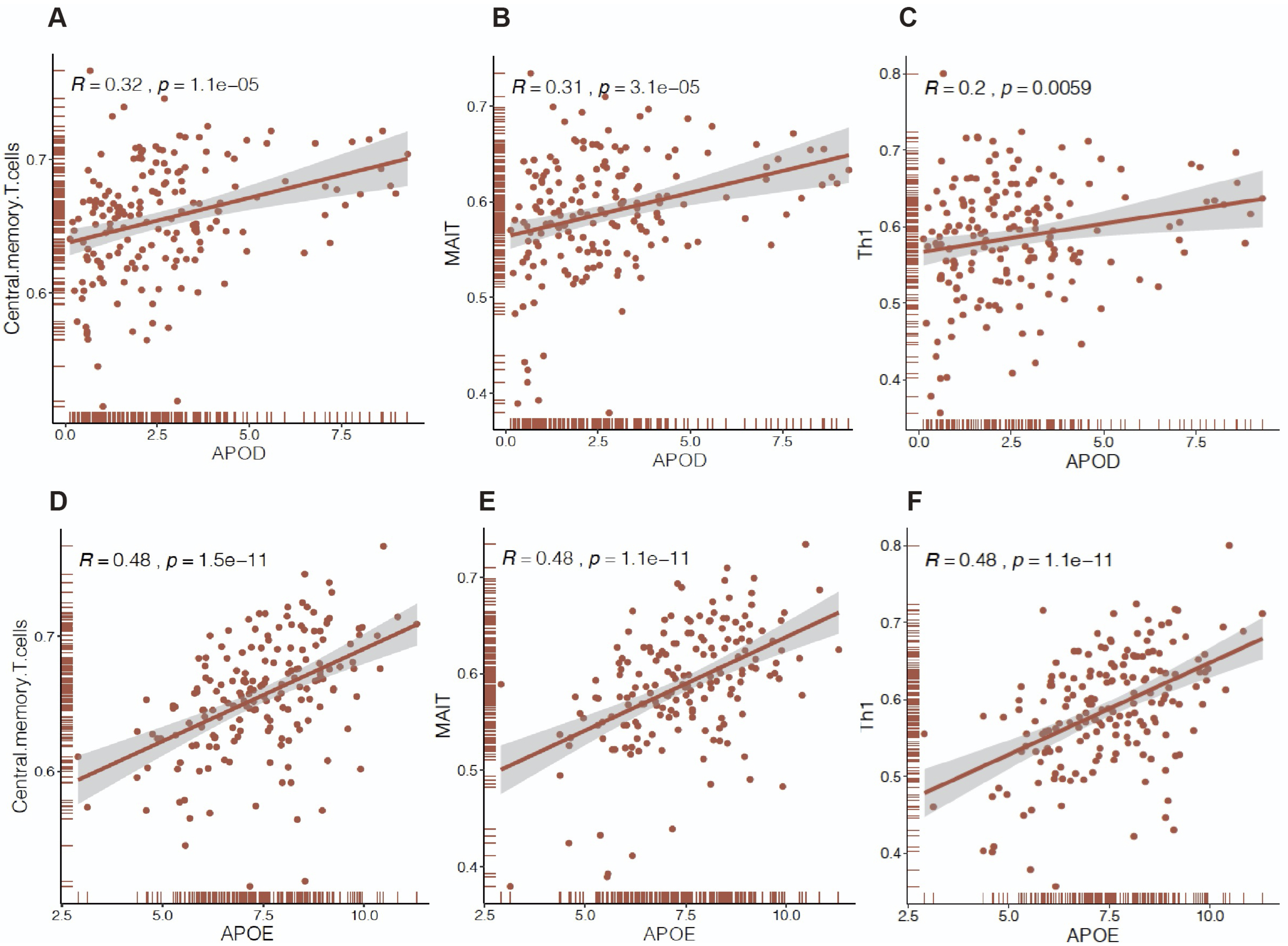

Supplement: Supplementary Figure 4 — Some immune infiltration cells correlation with APOD or APOE in EC by ssGESA. APOD or APOE expression were positively correlated the tumor infiltrating immune cells of Central Memory T cells (A, D), MAIT (Mucosal Associated Invariant T, MAIT) (B, E) and Th1(C, F). [file Image4.jpeg]

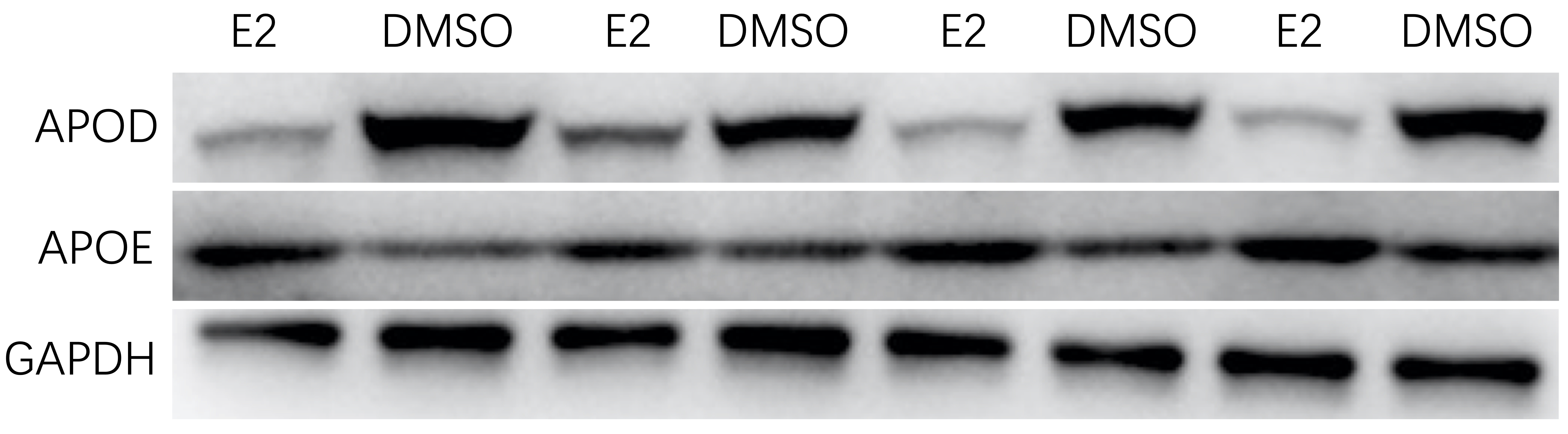

Supplement: Supplementary Figure 5 — Uncropped original Western blotting images. [file Image5.jpeg]
