# Supplementary material for: APOs as promising prognostic biomarkers: correlation with tumor-infiltrating leukocytes in endometrial cancer
Source: Front Immunol. 2026 Feb 16;17:1646920. doi: 10.3389/fimmu.2026.1646920 (PMC12950738; doi:10.3389/fimmu.2026.1646920)
Supplement: Supplementary file 6 [file Table1.docx]

Table S1 Antibodies for flowcytometry

| **Marker** | **Isotype** | **Clone number** | **Conjugation** | **Company** | **Cat No.** |
| --- | --- | --- | --- | --- | --- |
| CD3 | Mouse  (BALB/c) IgG1, κ | UCHT1 | V450 | BD | 560365 |
| CD4 | Mouse (BALB/c) IgG1, κ | SK3 | FITC | BD | 340133 |
| CD8 | Mouse IgG1, κ | SK1 | APC-Cy7 | BD | 663521 |
| CD19 | Mouse IgG1, κ | SJ25C1 | FITC | BD | 340409 |
| CD25 | Mouse IgG1, κ | 2A3 | PE | BD | 652834 |
| CD28 | Mouse (C3H x BALB/c) IgG1, κ | CD28.2 | PerCP-Cy™5.5 | BD | 560685 |
| CD29 | Mouse (BALB/c) IgG1, κ | 4-Mar | APC | BD | 559883 |
| CD45 | Mouse IgG1, κ | HI30 | V500 | BD | 560777 |
| CD45RA | Mouse (BALB/c) IgG1, κ | L48 | PE-Cy7 | BD | 649457 |
| CD45RO | Mouse (BALB/c) IgG2a, κ | UCHL-1 | PE | BD | 663530 |
| CD56 | Mouse IgG2b, κ | NCAM16.2 | PE-Cy7 | BD | 663487 |
| CD127 | Mouse IgG1, κ | HIL-7R-M21 | Alexa Fluor® 647 | BD | 558598 |
| HLA-DR | Mouse IgG2a, κ | L243 | APC-H7 | BD | 662909 |
