# Supplementary material for: APOs as promising prognostic biomarkers: correlation with tumor-infiltrating leukocytes in endometrial cancer
Source: Front Immunol. 2026 Feb 16;17:1646920. doi: 10.3389/fimmu.2026.1646920 (PMC12950738; doi:10.3389/fimmu.2026.1646920)
Supplement: Supplementary file 7 [file Table2.docx]

Table S2 Immune cells and their markers in flowcytometry

| Markers | Immune cells |
| --- | --- |
| CD3+ | CD3+ T cells, Total T cells |
| CD3-56+ | CD3NK cells |
| CD3-CD19+ | B cells |
| CD4+CD25+CD127- | natural Treg |
| CD3+CD4-CD8+ | CD8+ T cells, |
| CD3+CD4+CD8- | CD4+ T cells |
| CD3+CD8+CD28+ | Cytotoxic T cells, Tc |
| CD3+CD8+CD28- | Suppressive T cells, Ts |
| CD4+CD45RO+ | CD4+ Memory T cells |
| CD8+CD45RO+ | CD8+ Memory T cells |
| CD4+CD45RA+ | Naïve CD4+ T cells |
| CD8+CD45RA+ | Naïve CD8+ T cells |
| CD4+CD29+ | CD4+ effector T cells |
| CD3+HLA-DR+ | Activated T cells |
